# Case Report: Skeletal Muscle Lymphoma as a Result of Slow Centrifugal Migration of Untreated Primary Neurolymphomatosis?

**DOI:** 10.3389/fnume.2022.804421

**Published:** 2022-02-16

**Authors:** Sona Balogova, Radoslav Greksak, Magdalena Mizickova, Lucia Noskovicova, Pavel Babal, Ludovit Lukac

**Affiliations:** ^1^Department of Nuclear medicine, Faculty of Medicine, Comenius University in Bratislava and St. Elisabeth Oncology Institute, Bratislava, Slovakia; ^2^Nuclear Medicine, GH Tenon-St Antoine and Sorbonne University, Assistance Publique des Hôpitaux de Paris, Paris, France; ^3^Department of Haematology and Oncohaematology, Faculty of Medicine, Comenius University in Bratislava and National Oncology Institute, Bratislava, Slovakia; ^4^1^st^ Department of Radiology, Faculty of Medicine, Comenius University in Bratislava, Bratislava, Slovakia; ^5^Department of Pathological Anatomy, Faculty of Medicine, Comenius University in Bratislava, Bratislava, Slovakia; ^6^1^st^ Department of Internal Medicine, Comenius University in Bratislava, Bratislava, Slovakia

**Keywords:** FDG PET/CT, neurolymphomatosis (NL), skeletal muscle lymphoma, perineural invasion (PNI), centrifugal migration of neurolymphomatosis

## Abstract

**Introduction:**

Fludeoxyglucose (^18^F) (FDG) hybrid positron emission tomography/computed tomography (PET/CT) is currently a well-documented tool for diagnosis, staging, and therapeutic follow-up of lymphoma with significant impact on therapeutic decisions.

**Patient Concerns and Interventions:**

We reported a case of a 71-year-old woman with diffuse large B-cell lymphoma (DLBCL) of the left gluteal muscles as a possible result of slow centrifugal migration of untreated neurolymphomatosis (NL) of the lumbosacral plexus suggested on FDG PET/CT 4 years ago, when the patient was complaining for weakness and numbness of the left leg, but the proposed biopsy of peripheral nerve was not performed. Four years later, no pathological FDG uptake was present in nerves and lymph nodes, but PET/CT detected multiple FDG-positive infiltrates in the left gluteal muscles, appearing as a continuation of previously involved nerves.

**Diagnosis:**

The biopsy of muscular infiltrates confirmed DLBCL.

**Outcomes:**

The therapy was started, and a complete remission was achieved after three lines of treatment.

**Conclusion:**

This case contributes to limited knowledge on development of skeletal muscle lymphoma (SML): It suggests the macroscopically isolated, FDG-positive SML involving more than one muscular compartment as a possible consequence of natural course of untreated primary NL previously revealed by peripheral neuropathy and suspected on FDG PET/CT. This observation further justifies the consideration of implementation of FDG PET/CT into diagnostic algorithm while evaluating the peripheral neuropathy, in which the NL, albeit rare, is a part of differential diagnosis.

## Introduction

Neurolymphomatosis (NL) refers to direct infiltration of endoneurium by neoplastic cells and must be distinguished from direct compression of nerves due to adjacent lymphadenopathy or extranodal lymphomatous masses, as well as paraneoplastic neuropathies (1). Primary NL occurs as the initial and only manifestation of lymphoma at the time of diagnosis or with nodal or other extranodal manifestations. Clinically, it manifests as painful neuropathy or polyradiculopathy, painless polyneuropathy, cranial neuropathy, and peripheral mononeuropathy. Unlikely as in the case of meningoradicular spread of lymphoma, NL is typically confined to the peripheral nervous system and sparing the cerebrospinal fluid (2). Nevertheless, the overlapping cases were reported as well (3). The reason for typical central vs. peripheral compartmentalization remains speculative, but the type of lymphoma, site of the lymphoma in relation to the nervous system structures, and perhaps yet unknown properties of cell surface and migration ability might have some influence (2).

The increased use of Fludeoxyglucose (^18^F) (FDG) positron emission tomography/computed tomography (PET/CT) as a well-documented tool for diagnosis, initial staging, and therapeutic follow-up of lymphoma as well as increased use of MRI resulted in improved recognition of NL. With current treatments, the long-term survival of NL appears to have improved; however, no standard-of-care treatment exists, and the treatment needs to be individualized (4). The natural course of untreated primary NL is not known. The migration ability of lymphoma cells was suggested (2), but slow centrifugal migration of untreated NL into skeletal muscles with regression in peripheral nerves has never been reported so far. In fact, perineural invasion (PNI) is widely recognized as an important adverse pathological feature of many malignancies (5, 6).

Primary skeletal muscle lymphoma (SML) is even rarer condition than NL of unclear pathogenesis. In case of SML, no observations on direction (i.e., centripetal or centrifugal) of PNI are available; however, such information could contribute to better understanding of its pathogenesis.

We report the first case of SML as a possible result of slow centrifugal migration of untreated NL previously suspected on FDG PET/CT.

## Visit Summaries

### FDG PET/CT 1

The patient was referred to FDG PET/CT in the context of inflammatory syndrome and subfebrilities for localization of inflammatory, infectious, or solid malignant lesion(s) as the origin of symptoms.

**Figure 1:**
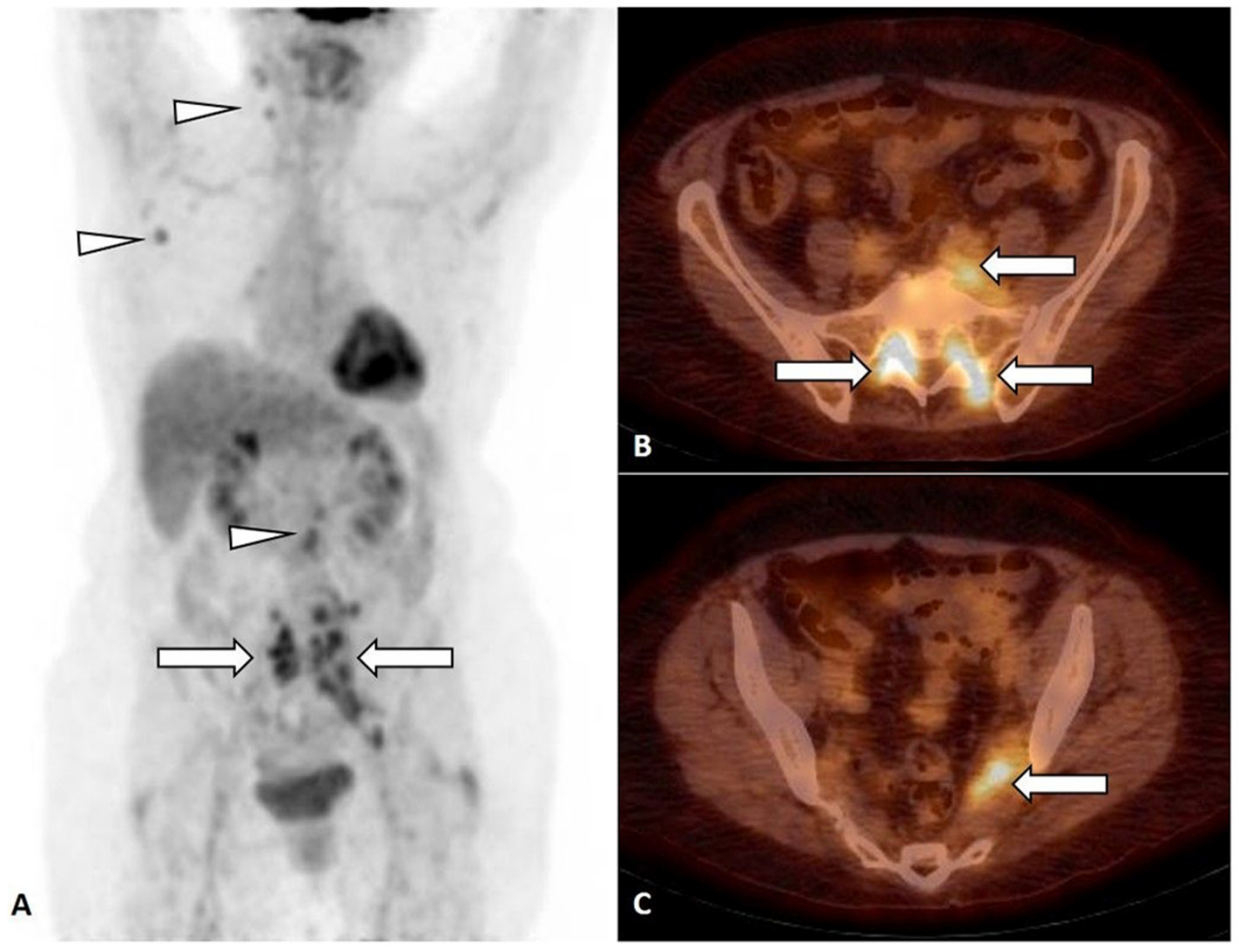
FDG PET, maximum intensity projection **(**MIP**) (A)** and FDG PET/CT, axial slice **(B,C)**. Focal FDG uptake in lumbosacral plexus (LSP), predominantly on the left side [**(A–C)**, arrow], in cervical, axillary, and retroperitoneal lymph nodes [**(A)**, arrowhead].

On PET/CT, an increased FDG uptake along lumbosacral plexus (LSP), predominantly on the left side (Figure 1A, arrow), in cervical, axillary, and retroperitoneal lymph nodes (Figure 1A, arrowhead) was observed. The NL of predominantly left LSP was suspected (Figures 1B,C, arrow), but biopsy was not performed.

### FDG PET/CT 2

Four years later, the patient was referred to the second FDG PET/CT in the context of inflammatory syndrome and subfebrilities non-responding to antibiotic treatment.

**Figure 2:**
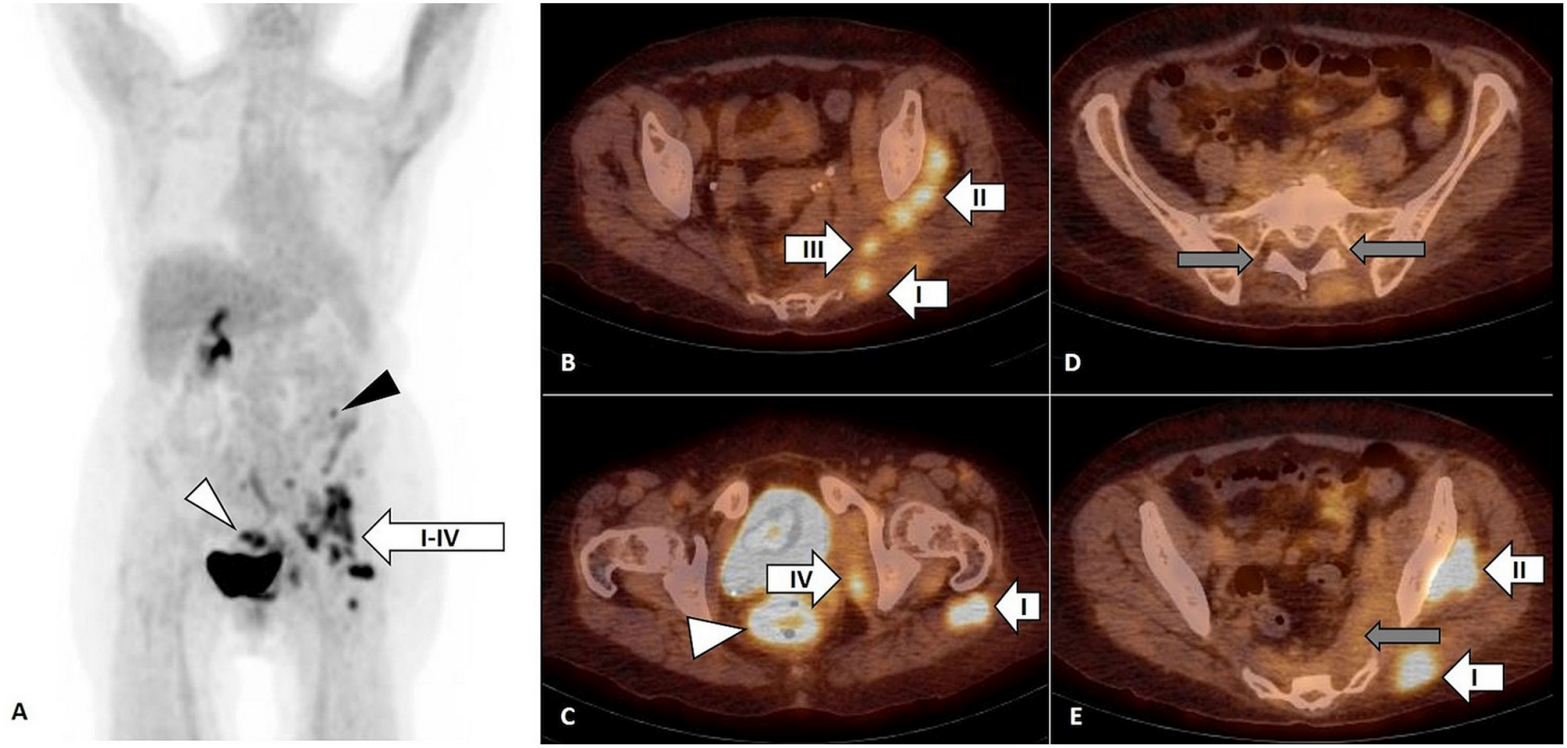
FDG PET, maximum intensity projection (MIP) **(A)** and FDG PET/CT, axial slice **(B–E)**. Focal FDG uptake by infiltrates in left gluteus maximus (I), medius (II), piriformis (III), and internal obturator (IV) muscles [**(A–C,E)**, arrow] confirmed by biopsy as diffuse large B-cell lymphoma (DLBCL). The FDG uptake in descending colon [**(A)**, black arrowhead] had no correlate on subsequent colonoscopy and the FDG uptake in rectum [**(A,C)**, white arrowhead] was related to pseudomembranous colitis as a complication of previous antibiotic treatment. The FDG uptake by lumbosacral plexus observed on previous FDG PET/CT was not present anymore [**(D,E)**, gray arrow].

On PET/CT, an increased FDG uptake by infiltrates in left gluteus maximus (I), medius (II), piriformis (III), and internal obturator (IV) muscles (Figures 2A–C,E, arrow), in descending colon (Figure 2A, black arrowhead) and in rectum (Figures 2A,C, white arrowhead). Inversely, the previously observed FDG uptake by LSP disappeared (Figures 2D,E, gray arrow)

## Narrative

FDG PET/CT (Figure 1) was performed in a 71-year-old woman with inflammatory syndrome, complaining for weakness and numbness of the left leg and showed increased FDG uptake in LSP, predominantly on the left side (Figure 1A, arrow), in cervical, axillary, and retroperitoneal lymph nodes (Figure 1A, arrowhead).

The NL of predominantly left LSP was suspected (Figures 1B,C, arrow), but, due to difficult accessibility and the risk of permanent neurological deficit, the biopsy of the nerve was not performed. The biopsy of non-enlarged FDG-positive cervical, axillary, and retroperitoneal lymph nodes was not performed. The stage IV lymphoma with NL of LSP with possible lymph node involvement was suggested, but no specific treatment was started.

During subsequent follow-up, the patient was treated by radiation therapy for painful arthrosis of the left knee with partial effect in terms of relief from pain. However, the weakness of the left leg persisted, which was attributed partly to pain.

Four years later, the second PET/CT was performed (Figure 2) in the context of inflammatory syndrome and subfebrilities non-responding to antibiotic treatment and showed focal FDG uptake by infiltrates in left gluteus maximus (I), medius (II), piriformis (III), and internal obturator (IV) muscles (Figures 2A–C,E arrow), in descending colon (2A, black arrowhead) and in rectum (Figures 2A,C, white arrowhead).

The FDG uptake in cervical, axillary, and retroperitoneal lymph nodes as well as in LSP observed on first PET/CT was not present on the second FDG PET/CT.

The colonoscopy to terminal ileum was performed with no endoscopic correlate of FDG-positive focus in descending colon. The biopsies of rectal and gluteal lesions were performed. The rectal lesion was concluded as pseudomembranous colitis as a complication of previous antibiotic treatment and targeted treatment was initiated.

**Figure 3:**
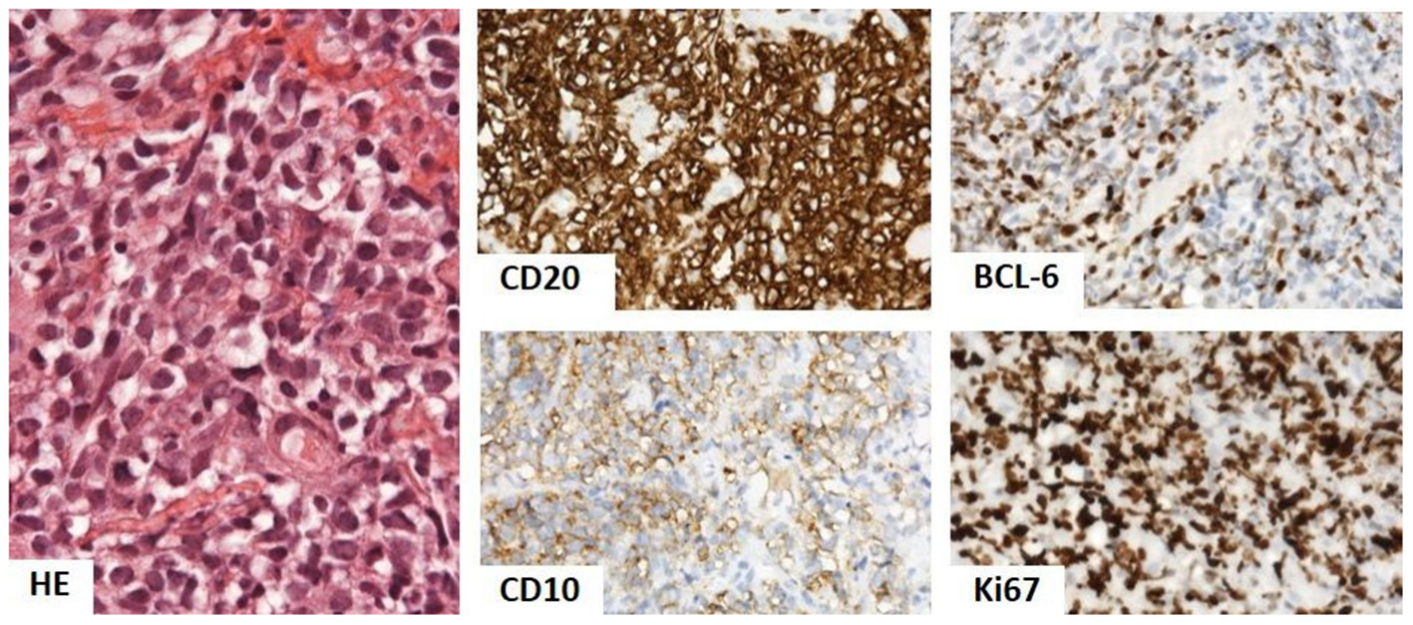
Biopsy of gluteal mass showed dense infiltrate of oval lymphoid cells with light eosinophilic cytoplasm and oval nucleus with prominent indentation and conspicuous 1–3 nucleoli. The phenotype was of B-cells (CD20), with CD10 positivity, BCL-6 in over 50%, and Ki67 in over 90%, corresponding to diffuse large B-cell lymphoma (DLBCL), germinal center-B phenotype, and anaplastic variant. Hematoxylin and eosin (HE) stain 400×, immunoperoxidase technique, diaminobenzidine 200×.

The biopsy of gluteal mass (Figure 3) was consistent with diffuse large B-cell lymphoma (DLBCL), germinal center-B phenotype, anaplastic variant. Complete remission was achieved after three lines of oncological treatment.

## Diagnostics

Biopsy of gluteal mass: DLBCL, germinal center-B phenotype, and anaplastic variant.

## Discussion

DLBCL is an aggressive disease corresponding to more than 30% of all lymphomas. Approximately 30% of DLBCL correspond to extranodal forms involving the most frequently small intestine, mediastinum, thyroid, adrenal, breast, uterine, kidney, and testis (7).

Without treatment, the prognosis of DLBCL is poor in majority of cases; the rare spontaneous remissions have been described during up to 42 months after diagnosis (8); however, as the treatment of DLBCL usually starts soon after diagnosis, the understanding of mechanisms leading to its spontaneous remission is limited.

In present case, neither the biopsy of suspected FDG-positive NL of predominantly left LSP nor the biopsy of FDG-positive non-enlarged cervical, axillary, or retroperitoneal lymph nodes was performed, which only permits to suggest the lymphoma as an underlying pathology on the basis of FDG PET/CT result, clinical follow-up, and biopsy.

The differential diagnosis of FDG-positive lymph nodes is wide, but, regardless of their etiology, the multiple extranodal involvement of peripheral nerves permitted to classify the suggested lymphoma as stage IV of the disease.

The unusual aspect of the present case is the spontaneous evolution of suspected untreated NL of the left LSP.

Primary NL is a separate entity from central nervous system lymphoma and has most commonly been described in association with B-cell non-Hodgkin lymphoma. It is a rare condition representing ~5% of all lymphomas (9); however, while evaluating peripheral neuropathy, a high degree of suspicion of NL is required because the presenting symptoms may vary and a pathological diagnosis is often difficult. As confirmed by observations dated from the era before extensive use of functional imaging with FDG PET/CT, primary NL is frequently undiagnosed, with 45% of patients diagnosed as NL only after autopsy (10).

Concerning the anatomical localization of primary NL, isolated involvement of LSP is the first clinical manifestation of lymphoma in 74% cases of NL, with sciatic localization in all cases (9). The biopsy is often difficult to perform because not all of the nerves are accessible in a biopsy procedure and there is a possibility of a permanent large deficit, which probably contributes to underestimation of data on incidence of primary NL.

Functional imaging using FDG PET/CT is currently the most sensitive and specific whole-body imaging technique for lymphoma (11). High sensitivity of FDG PET/CT for NL with significant impact on diagnostic thinking and on therapeutic management was suggested by several case reports (12–18). In a series of five patients, Shree et al. (11) reported the 100% sensitivity of FDG PET/CT for detection of NL. Nevertheless, one case of false negative finding of FDG PET/CT in a patient with T-cell lymphoma and NL was reported (2). When comparing with morphological imaging methods, in early detection of peripheral nerve involvement in NL, the FDG PET/CT was reported to be more sensitive than MRI (12, 18) probably also due to whole-body character of FDG PET/CT imaging.

In this context, it is possible that, with cumulated experience and with current use of FDG PET/CT, the figures of incidence of NL will increase.

Concerning the specificity of FDG PET/CT for NL, to our best knowledge, the false-positive findings for NL due to inflammatory or infectious processes involving peripheral nerves or in case of chronic inflammatory demyelinating polyneuropathy were not reported.

In the present patient, the persistent weakness of the left leg was partly attributed to painful arthrosis of the left knee.

Four years later, on the second FDG PET/CT performed for inflammatory syndrome and subfebrilities non-responding to antibiotic treatment, the FDG uptake along LSP or in lymph nodes was not present anymore, but several FDG-positive infiltrates in the left gluteal, piriformis, and obturator muscles occurred and were confirmed by biopsy as DLBCL. The analysis of these two FDG PET/CT findings supported by bibliographic data on high positive predictive value of FDG PET/CT for NL led to hypothesis of SML as a possible result of slow perineural centrifugal migration of previously detected untreated NL within little understood processes of interactions between cancer and host.

The FDG uptake by cervical, axillary, and retroperitoneal lymph nodes observed on the first FDG PET/CT was not present on the second FDG PET/CT, suggesting either absence of their relation with later confirmed DLBCL or spontaneous remission of lymphoma in lymph nodes, which is extremely rare (particularly in stage IV DLBCL), but described in the literature (8). The rare cases of spontaneous remission of DLBCL were reported mainly in patients after biopsy, suggesting that trauma may trigger the processes, leading to spontaneous remission of DLBCL. In the present patient, the radiation therapy for painful arthrosis of the left knee was performed after the first FDG PET/CT, but the abscopal effect leading to remission of lymphomatous nodal involvement is extremely improbable.

The spontaneous remission of NL with or without perineural migration to peripheral skeletal muscles has never been described so far.

The primary SML is even less frequent condition than primary NL (19) with reported frequency varying from 0.1 to 1.4% of all extranodal lymphomas (20–22). Among all cases of malignant muscle tumors, SML is estimated to represent 1.2 to 2.0% of all cases (23).

The SML is usually presenting with muscle pain and swelling located in lower limbs in majority of cases (19). The pathogenesis of SML is unclear. It has been proposed that SML can originate in aberrant lymph nodes within muscles (24, 25), and, therefore, the term “extranodal” may be misleading in this context. Some authors describe lymphomatous microinfiltrate originating from the adjacent bone as possible cause of SML (26).

In the present case, we suggested the SML as a possible result of slow centrifugal perineural migration of untreated NL, which has never been proposed so far. PNI is widely recognized as an important adverse pathological feature of many malignancies. Despite the widespread acknowledgment of the clinical significance of PNI, the mechanisms underlying its pathogenesis remain unknown. Recent theories of PNI pathogenesis place the great emphasis on the active role of the nerve microenvironment, with PNI resulting from well orchestrated reciprocal interactions between cancer and host. Although traditionally viewed as a cancer driven process, PNI seems to be equally facilitated by the host. There is reciprocity in interactions at cancer and nerve interface, resulting in a migratory and growth advantages for both the cancer and the nerve (5). The PNI typically spreads centripetally; nevertheless, the centrifugal spread is not uncommonly seen, e.g., in head and neck cancer (6).

The data on centrifugal spread of NL are not available. The hypothesis of SML as a result of perineural centrifugal migration of untreated NL is supported by two cases of simultaneous nervous and muscular involvement; in both of them, the muscular involvement was preceded by neurological symptoms (27, 28).

In one case, the SML was manifested as trigeminal neuralgia and the lymphoma mass was later detected in external pterygoid muscles and brainstem through swelled trigeminal nerve (28). In the second case, the SML of the tongue was first manifested as trigeminal neuralgia followed by development of painless tongue mass (27); in this case, on PET/CT, the FDG uptake was observed in the left Meckel's cave and in the left side tongue mass (27).

In both cases (27, 28), the treatment was initiated soon after diagnosis, and it is therefore not possible to estimate the spontaneous evolution of neural involvement; nevertheless, these two cases, together with the present case, suggest the centrifugal migration of NL with (27, 28) or without (present case) concomitant neural and skeletal muscular involvement as a possible mechanism involved in development of SML.

Moreover, according to MRI studies, the extension along neurovascular structures (29, 30) observed in 29% of cases together with involvement of more than one muscular compartment are considered typical features of SML (31). These one–time point observations of Suresh et al. (31) with no data on presence or absence of eventual neurological symptoms preceding the diagnosis of SML do not permit to unequivocally conclude the direction of PNI observed in SML patients on MRI (31); however, the involvement of more than one muscular compartment (which is unusual for sarcomas) may be one of the elements supporting the hypothesis of SML as a result of PNI.

## Conclusion

The present case suggests the SML as a result of slow centrifugal migration of untreated primary NL revealed by peripheral neuropathy and suspected on FDG PET/CT and contributes to limited knowledge on development of this entity.

The mechanism of spontaneous clearance of lymphomatous cells from peripheral nerves toward skeletal muscles is, the most probably, a part of little understood processes of interaction between cancer and host, and their mechanisms as well as clinical significance need to be analyzed.

Moreover, the present observation further justifies the consideration of implementation of FDG PET/CT into diagnostic algorithm while evaluating the peripheral neuropathy, in which the NL, albeit rare, is a part of differential diagnosis and highlights the need for histological verification of pathological FDG uptake by peripheral nerves.

## Data Availability Statement

The original contributions presented in the study are included in the article, further inquiries can be directed to the corresponding author.

## Ethics Statement

Ethical review and approval was not required for the study on human participants in accordance with the local legislation and institutional requirements. The patient provided their written informed consent to participate in this study.

## Author Contributions

SB: manuscript writing and editing and manuscript approval. RG: patient's referral and follow-up and manuscript approval. MM: CT, biopsy, and manuscript approval. LN: PET/CT and manuscript approval. PB: histopathology, manuscript editing, and manuscript approval. LL: patient's referral and follow-up, manuscript editing, and manuscript approval.

## Funding

This study was funded by KEGA 058UK-4/2020.

## Conflict of Interest

The authors declare that the research was conducted in the absence of any commercial or financial relationships that could be construed as a potential conflict of interest.

## Publisher's Note

All claims expressed in this article are solely those of the authors and do not necessarily represent those of their affiliated organizations, or those of the publisher, the editors and the reviewers. Any product that may be evaluated in this article, or claim that may be made by its manufacturer, is not guaranteed or endorsed by the publisher.

## Abbreviations

DLBCL: Diffuse large B-cell lymphoma
FDG: Fludeoxyglucose (^18^F)
LSP: Lumbosacral plexus
MRI: Magnetic resonance imaging
NL: Neurolymphomatosis
PET/CT: Hybrid positron emission tomography/computed tomography
PNI: Perineural invasion
SML: Skeletal muscle lymphoma.
